# Association of Repetitive Transcranial Magnetic Stimulation Treatment With Subgenual Cingulate Hyperactivity in Patients With Major Depressive Disorder: A Secondary Analysis of a Randomized Clinical Trial

**DOI:** 10.1001/jamanetworkopen.2019.5578

**Published:** 2019-06-05

**Authors:** Itay Hadas, Yinming Sun, Pantelis Lioumis, Reza Zomorrodi, Brett Jones, Daphne Voineskos, Jonathan Downar, Paul B. Fitzgerald, Daniel M. Blumberger, Zafiris J. Daskalakis

**Affiliations:** 1 Temerty Centre for Therapeutic Brain Intervention, Centre for Addiction and Mental Health, University of Toronto, Toronto, Ontario, Canada; 2 Department of Psychiatry and Behavioral Sciences, Stanford University School of Medicine, Stanford, California; 3 MRI-Guided rTMS Clinic, Toronto, Ontario, Canada; 4 Krembil Research Institute, Toronto, Ontario, Canada; 5 Department of Psychiatry, Faculty of Medicine, University of Toronto, Toronto, Ontario, Canada; 6 Institute of Medical Science, University of Toronto, Toronto, Ontario, Canada; 7 Epworth Centre for Innovation in Mental Health, Epworth HealthCare, Camberwell, Victoria, Australia; 8 Monash Alfred Psychiatry Research Centre, Monash University Central Clinical School, Melbourne, Victoria, Australia

## Abstract

**Importance:**

Hyperactivity in the subgenual cingulate cortex (SGC) is associated with major depressive disorder (MDD) and anticorrelated with activity in the dorsolateral prefrontal cortex (DLPFC). This association was found to be predictive of responsiveness to repetitive transcranial magnetic stimulation (rTMS) treatment. Such findings suggest that DLPFC-SGC connectivity is important for understanding both the therapeutic mechanism of rTMS in patients with MDD and the underlying pathophysiology of MDD.

**Objective:**

To evaluate SGC hyperactivity in patients with MDD before and after rTMS treatment.

**Design, Setting, and Participants:**

In this diagnostic study, among participants recruited from the adult and geriatric mood and anxiety services at the Centre for Addiction and Mental Health, Toronto, Ontario, Canada, who had participated in a randomized clinical trial, baseline SGC activity of patients with MDD was compared with healthy controls. In patients with MDD, SGC activity was compared before and after active or sham high-frequency rTMS treatment. Data collection started in July 2008 and concluded in March 2012. Neurophysiological data analysis started in January 2017 and ended in May 2018.

**Main Outcomes and Measures:**

Hyperactivity in the SGC before and after rTMS treatment was measured. Subgenual cingulate cortex hyperactivity activity was quantified using significant current density (SCD), and effective connectivity between the left DLPFC and SGC was computed using significant current scattering (SCS). Both measures were computed around TMS evoked potentials standard peak latencies prior to rTMS and after rTMS treatment, comparing patients with MMD treated with active and sham rTMS. Patients with MDD were assessed with the 17-item Hamilton Rating Scale for Depression.

**Results:**

Of 121 patients with MDD in the initial trial, 30 (15 [50.0%] women) were compared with 30 healthy controls (15 [50.0%] women) at rTMS treatment baseline. The mean (SD) age of the cohort with MDD was 39.1 (10.9) years, and the mean (SD) age of healthy controls was 37.0 (11.0) years. Following rTMS treatment, 26 patients with MDD who had active rTMS treatment (21.5%) were compared with 17 patients with MDD who had sham treatment (14.0%). At baseline, the SGC mean (SD) SCD and mean (SD) SCS at 200 milliseconds after TMS pulse were higher in participants with MDD compared with healthy controls (SCD: 1.04 × 10^−6^ [1.41 × 10^−6^] μA/mm^2^ vs 3.8 × 10^−7^ [7.8 × 10^−7^] μA/mm^2^; *z* = –2.95; *P* = .004; SCS: 0.87 [0.86] mm vs 0.54 [0.87] mm; *z* = –2.27; *P* = .02). Baseline source current density was able to classify MDD with 77% accuracy. Scores on the 17-item Hamilton Rating Scale for Depression were correlated with current density at the SGC (ρ = 0.41; *P* = .03). After rTMS treatment, SGC mean (SD) SCD and mean (SD) SCS at 200 milliseconds after rTMS pulse were attenuated to approximately the standard TMS-evoked potential latencies in the active rTMS group compared with the sham rTMS group (SCD: 1.57 × 10^−7^ [3.67 × 10^−7^] μA/mm^2^ vs 7.00 × 10^−7^ [7.51 × 10^−7^] μA/mm^2^; *z* = −2.91; *P* = .004; SCS: 0.20 [0.44] mm vs 0.74 [0.73] mm; *z* = −2.78; *P* = .006). Additionally, the SGC SCS change was correlated with symptom improvement on the 17-item Hamilton Rating Scale for Depression in the active rTMS group (ρ = 0.58; *P* = .047).

**Conclusions and Relevance:**

The findings of this study further implicate left DLPFC-SGC effective connectivity and SGC excitability in the pathophysiology of MDD and treatment with rTMS. These findings suggest that DLPFC-SGC connectivity may be a marker of rTMS treatment responsiveness.

**Trial Registration:**

ClinicalTrials.gov identifier: NCT01515215

## Key Points

**Question:**

Is repetitive transcranial magnetic stimulation associated with changes in subgenual cingulate cortex (SGC) activity in patients with major depressive disorder (MDD)?

**Findings:**

This diagnostic study, which compared 30 patients with MDD and 30 healthy controls, found that using transcranial magnetic stimulation combined with electroencephalography, SGC activity in patients with MDD was significantly higher compared with healthy controls. After active repetitive transcranial magnetic stimulation, SGC hyperactivity in patients with MDD was attenuated toward the levels of healthy controls.

**Meaning:**

These SGC-localized findings support SGC hyperactivity as a central construct in the pathophysiology of MDD, which future work might develop into a clinically significant biological target.

## Teaser

This diagnostic study evaluates subgenual cingulate cortex hyperactivity in patients with major depressive disorder before and after repetitive transcranial magnetic stimulation treatment compared with a group of healthy controls.

## Introduction

Major depressive disorder (MDD) is a debilitating psychiatric condition with a 16% lifelong prevalence.^1^ Antidepressant medication is widely used and has been researched for more than 4 decades. However, the mechanism of antidepressant action remains unclear, and nearly 40% of patients with MDD experience persistent depression even after 2 antidepressant treatments.^2^ To improve outcomes, the neurophysiology of MDD and underlying therapeutic mechanisms of treatments need to be better understood.

Hyperactivity of the subgenual cingulate cortex (SGC) is associated with MDD pathophysiology. Based on positron emission tomography studies,^3^ the SGC was shown to be overactive in the depressed state and during transient sadness in healthy controls. Furthermore, higher SGC activity was also found in patients with MDD in both depressed and remitted states.^3^ The SGC has also been a primary target for deep brain stimulation in the treatment of MDD.^4^

Evidence also implicates dorsolateral prefrontal cortex (DLPFC)–SGC connectivity in MDD pathophysiology. An inverse association of the SGC with right DLPFC activity was found in patients with MDD and healthy patients,^3^ implying that there is a functional association of the SGC with the DLPFC. Moreover, functional magnetic resonance imaging showed that DLPFC-SGC activity is anticorrelated,^5^ and the magnitude of this anticorrelation can predict the antidepressant efficacy of repetitive transcranial magnetic stimulation (rTMS) targeting the DLPFC. Finally, both positron emission tomography and functional magnetic resonance imaging studies have shown that baseline SGC activation abnormalities were attenuated toward healthy levels using a variety of antidepressant treatments, including conventional antidepressant medications,^6,7,8,9^ rTMS,^10,11^ electroconvulsive therapy,^12^ and deep brain stimulation.^4^

Combining TMS with electroencephalography (TMS-EEG) is a noninvasive technique that is used to assess neuronal activity,^13,14^ connectivity,^15,16^ and plasticity.^17^ Compared with other neurophysiological methods, TMS-EEG enables a more reliable and causal assessment of the neurophysiology of brain function. This is owing to the fact that TMS-EEG stimulates the cortex directly without dependence on prior activation of upstream brain functional regions (eg, sensory constructs).^16^ Significant current density (SCD) is a standardization measure that sums all of the significant TMS-responsive current sources (compared with baseline). Significant current scattering (SCS) is a standardization measure for inferring activation propagation; SCS may also measure the effective connectivity of a source relative to the stimulation site.^13,18^ Previously, SCD has been computed to differentiate between patients with Alzheimer disease and healthy controls.^19^ The 2 measures have also been used to capture changes in brain network connectivity during loss of consciousness,^18^ saccadic movement,^20^ and task performance.^21^ Significant current scattering has also been effective in distinguishing patients with schizophrenia from healthy controls.^22^

We used TMS-EEG measures of SCD to evaluate SGC excitability and TMS-EEG measures of SCS to evaluate DLPFC-SGC effective connectivity in patients with MDD. These measures were taken at baseline and after rTMS treatment. We hypothesized that SCD would demonstrate higher excitability in the SGC of patients with MDD. We also hypothesized that SCS would show a stronger effective connectivity between the left DLPFC and the SGC in patients with MDD. Finally, we hypothesized that increased excitability in (ie, SCD) and connectivity to (ie, SCS) the SGC would be attenuated after applying rTMS over the DLPFC in patients with MDD.

## Methods

### Recruitment and Treatment

**Figure 1.**
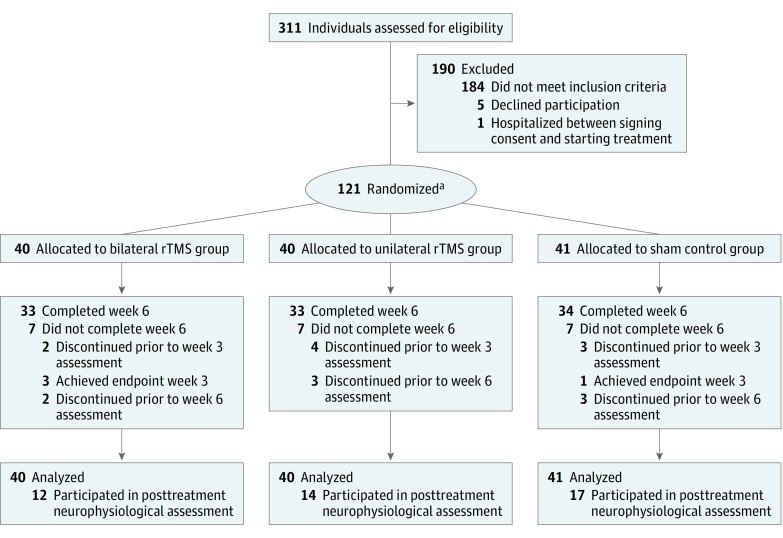
Flowchart of Participants in the Original Randomized Clinical Trial rTMS indicates repetitive transcranial magnetic stimulation. ^a^Of the 121 participants, 30 took part in a pretreatment neurophysiological assessment, the results of which were compared with assessments from 30 healthy controls.

Overall, 121 participants with MDD (77 [63.6%] women) were recruited at the Centre for Addiction and Mental Health in Toronto, Ontario, Canada. These patients participated in a randomized clinical trial, as previously described.^23^ The demographic and clinical characteristics are described in the eTable in the Supplement. The adverse effects of rTMS on study participants have been previously described.^23^ A random subset of participants in the randomized clinical trial (n = 30) underwent a pretreatment neurophysiological assessment. We recruited an additional 30 healthy controls separately for a neurophysiological measures comparison. Of 101 participants who completed week 6 of the trial, 43 underwent posttreatment neurophysiological assessments, had usable EEG recordings, and were analyzed for TMS-EEG neurophysiological effects. The participants with MDD were randomized to 3 different treatment arms: unilateral rTMS stimulation (n = 40), bilateral rTMS stimulation (n = 40), and sham stimulation (n = 41) (Figure 1). The rTMS protocol and parameters were conducted per the article by Blumberger et al.^23^ Neurophysiological measurements by TMS-EEG were performed 1 week prior to rTMS treatment and within 48 hours of the last rTMS treatment. The 17-item Hamilton Rating Scale for Depression (HRSD-17)^24^ was given to patients before and after rTMS treatment. Participants gave written informed consent, and the protocol was approved by the Centre for Addiction and Mental Health ethics board in accordance with Declaration of Helsinki.^25^ This report follows the Consolidated Standards of Reporting Trials (CONSORT) reporting guideline.

### Acquisition and Preprocessing of TMS-EEG Data

In all groups, a figure-of-eight coil connected to Magstim 200 stimulators (Magstim) was used to stimulate the brain over the left DLPFC. Locating the motor cortex was done by eliciting motor-evoked potentials at the right abductor pollicis brevis. The left DLPFC was identified using the miniBIRD neuronavigation system (Ascension Technologies). Before each experiment, resting motor threshold was determined, as previously described.^26^ Each patient’s stimulus intensity was determined as the intensity eliciting peak-to-peak amplitude of 1 mV averaged over 20 trials. Electroencephalography was performed using a 64-channel Synamps 2 EEG system (Compumedics Neuroscan). It was recorded applying a 200-Hz low-pass filter at a 20-kHz sampling rate. All electrodes (silver/silver chloride ring) impedances were kept less than 5 kΩ throughout the session, and the ground electrode was positioned posteriorly to the Cz electrode. Electroencephalography analysis was performed using MATLAB version r2017b (The MathWorks) EEGLAB^27^ and FieldTrip^28^ toolboxes. Noisy channels were removed (with high-powered 60-Hz amplitudes or with extremely high and variable amplitudes). The EEG recording was epoched around the TMS pulse (1 second before and after). Transcranial magnetic stimulation pulses were removed from each epoch (−2 milliseconds to 20 milliseconds) and linearly interpolated. Noisy epochs were removed. The data were baseline-corrected (500 milliseconds to 200 milliseconds prepulse) and average-referenced. Data was resampled to 1 kHz. A first round of independent component analysis was used to remove large muscle artifacts. The data were bandpass filtered (1-100 Hz), with a 58- to 62-Hz notch. A second round of independent component analysis was used to remove eye blinks, eye movements, and additional muscle artifacts. Finally, missing electrodes were interpolated. Further details regarding our TMS-EEG preprocessing can be found in the article by Rogasch et al.^29^

### Source Localization Procedure

Source analysis of the TMS-evoked potential (TEP) was done using the MATLAB Brainstorm toolbox.^30^ A generic brain with 15 002 voxels, based on the Montreal Neurological Institute International Consortium for Brain Mapping 152–averaged magnetic resonance imaging for the extracted cortex surface,^31^ was used. The EEG cap used in the experiment, Neuroscan 64-channel quik-cap (Compumedics Neuroscan), was coregistered to the generic head model. The forward model was computed using the OpenMEEG approach with a solution space limited to the cortex surface.^32^ The prestimulus period of individual trials was used to calculate the noise covariance. Finally, the inverse solution was computed based on the standardized low-resolution brain electromagnetic tomography algorithm,^33^ with dipoles constrained normally to the cortex surface. For each patient, the source localization procedure generated a 15 002-voxel current density map in brain space for every point of the TEP.

Significant current scatter was calculated based on the following equation adapted from methods previously published in the article by Casali et al^13^: SCS = *SS(x, t) × d(x* *–* *x_stim_*), in which *SS(x, t)* is a binary matrix of significant sources across the brain at each point of the TEP and *d(x* *–* *x_stim_*) is calculated as the distance of every source voxel from site of stimulation (ie, F5). To calculate *SS(x, t)*, each poststimulus point trial distribution was compared by a paired *t* test with a surrogate distribution taken from a point in a prestimulus time segment. A current dipole at a specific point was considered significant if its 2-sided α was less than .05 in a paired *t* test compared with the surrogate responses at that time point.

To compare the overall scatter values from the TMS target (the DLPFC) to the SGC, SCS magnitudes were summed across the TEP peak standard time periods (approximately 30 milliseconds, 60 milliseconds, 100 milliseconds, and 200 milliseconds) for the right and left SGC regions as defined by the Destrieux atlas.^34^

### Statistical Analysis

Both bilateral and unilateral rTMS treatment groups were pooled together into the active rTMS treatment group owing to statistical power considerations. To avoid normality assumptions, the Wilcoxon rank sum test was used to examine the differences between current density, SCD, and SCS in patients with MDD and healthy controls and in patients with MDD in the different rTMS treatment arms. Statistical significance was set at *P* < .05, and all tests were 2-tailed. No correction for multiple comparisons was applied. A Spearman correlation was used to quantify the association of SCS change with HRSD-17 score change before and after treatment. Finally, a receiver operating characteristic analysis was computed for the pre-rTMS sample; a source current density amplitude window from 15 milliseconds to 350 milliseconds was used as the criterion to classify healthy controls vs patients with MDD. This time segment was chosen because significant sources dropped drastically 350 milliseconds after stimulation. The probability of correct prediction was quantified by the area under the receiver operating characteristic curve,^35^ while the optimal threshold was determined as the source current density value associated with the maximum Youden index, or height above the diagonal line of no discrimination.^36^ All statistical analysis was done using MATLAB version r2017b.

## Results

Overall, 30 of 121 trial participants with MDD (15 [50.0%] women) pre-rTMS treatment were compared with 30 healthy controls (15 [50.0%] women). The mean (SD) age of the cohort with MDD was 39.1 (10.9) years, and the mean (SD) HRSD-17 score was 24.8 (3.5). The mean (SD) age of the healthy controls was 37.0 (11.0) years (eTable in the Supplement). There was no age difference between the active and sham rTMS treatment groups (*t* = −0.47; *P* = .46).

**Figure 2.**
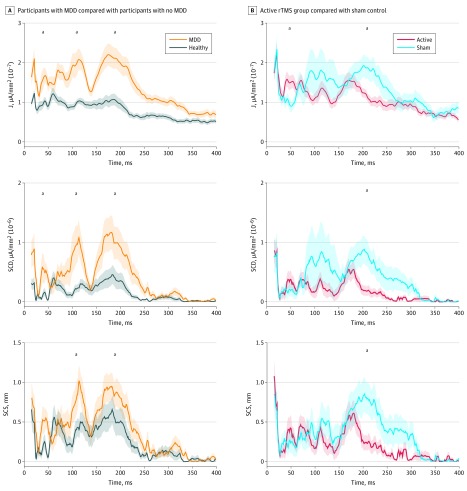
Current Density (J), Significant Current Density (SCD), and Significant Current Scattering (SCS) After Single Transcranial Magnetic Stimulation A, Participants with major depressive disorder (MDD) compared with healthy controls. B, Participants in active groups compared with participants in sham group. Shaded areas represent ±1 SEM. ^a^*P* < .05.

**Figure 3.**
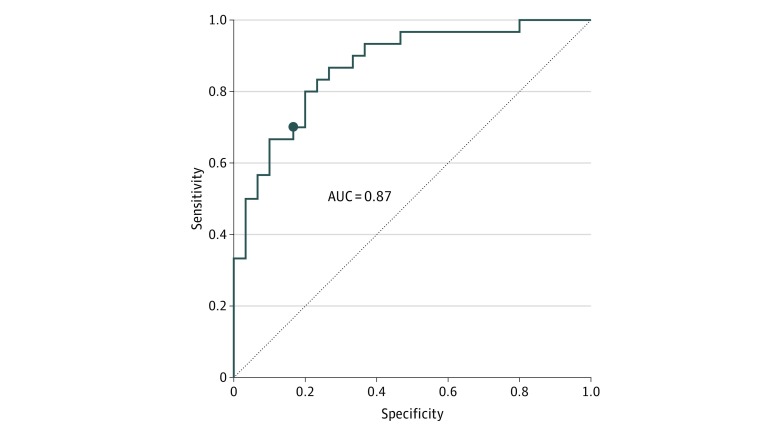
Receiver Operating Characteristic Curve Showing Classification Model Performance Model quality is demonstrated by the area under the curve (AUC). Based on Youden index, the optimal model (marked by dot) differentiates major depressive disorder from healthy control with 77% accuracy (70% sensitivity and 83% specificity).

Overall, 26 patients with MDD (21.5%; 17 [65.4%] women) who received active rTMS treatment were analyzed. They had a mean (SD) age of 47.3 (14.0) years and mean (SD) HRSD-17 score of 25.6 (2.9). We analyzed 17 patients with MDD (14.0%; 8 [47.1%] women) who received sham rTMS. They had a mean (SD) age of 45.7 (12.5) years and a mean (SD) HRSD-17 score of 25.3 (3.2) (eTable in the Supplement). There were no age differences between the active and sham rTMS treatment groups (*t* = 0.17; *P* = .70). Patients with MDD demonstrated significant differences on SCD and SCS computations compared with healthy controls (Figure 2A). These differences were localized in voxels in the region of the SGC, and their timings aligned with standard TEP temporal components (P30 milliseconds, N100 milliseconds, and P200 milliseconds). Mean (SD) current density in the SGC was higher at 30 milliseconds for patients with MDD compared with healthy controls (1.51 × 10^−7^ [7.18 × 10^−8^] μA/mm^2^ vs 9.42 × 10^−8^ [4.23 × 10^−8^] μA/mm^2^; *z* = −3.42; *P* < .001), at 100 milliseconds (2.05 × 10^−7^ [1.28 × 10^−7^] μA/mm^2^ vs 1.02 × 10^−7^ [4.7 × 10^−8^] μA/mm^2^; *z* = –4.13; *P* < .001), and at 200 milliseconds (2.1 × 10^−7^ [1.37 × 10^−7^] μA/mm^2^ vs 1.01 × 10^−7^ [7.45 × 10^−8^] μA/mm^2^; *z* = –4.26; *P* < .001) after the TMS pulse. Mean (SD) SCD in the SGC in patients with MDD compared with healthy controls was higher at 30 milliseconds (4.26 × 10^−7^ [5.04 × 10^−7^] μA/mm^2^ vs 1.12 × 10^−7^ [1.67 × 10^−7^] μA/mm^2^; *z* = –2.57; *P* = .01), at 100 milliseconds (9.43 × 10^−7^ [1.41 × 10^−6^] μA/mm^2^ vs 2.83 × 10^−7^ [4.47 × 10^−7^] μA/mm^2^; *z* = –2.55; *P* = .01), and at 200 milliseconds (1.04 × 10^−6^ [1.41 × 10^−6^] μA/mm^2^ vs 3.8 × 10^−7^ [7.8 × 10^−7^] μA/mm^2^; *z* = –2.95; *P* = .004) after the pulse. Additionally, the mean (SD) SCS between the stimulation site (left DLPFC) and SGC was higher in patients with MDD compared with healthy controls at 100 milliseconds (0.93 [0.99] mm vs 0.47 [0.66] mm; *z* = –1.97; *P* = .048) and at 200 milliseconds (0.87 [0.86] mm vs 0.54 [0.87] mm; *z* = –2.27; *P* = .02) after the TMS pulse. A receiver operating characteristic curve analysis was used to identify the predictive power of source current density in patients with MDD compared with healthy controls. The model differentiated patients with MDD from the healthy controls with 77% accuracy (70% sensitivity and 83% specificity) (Figure 3).

**Figure 4.**
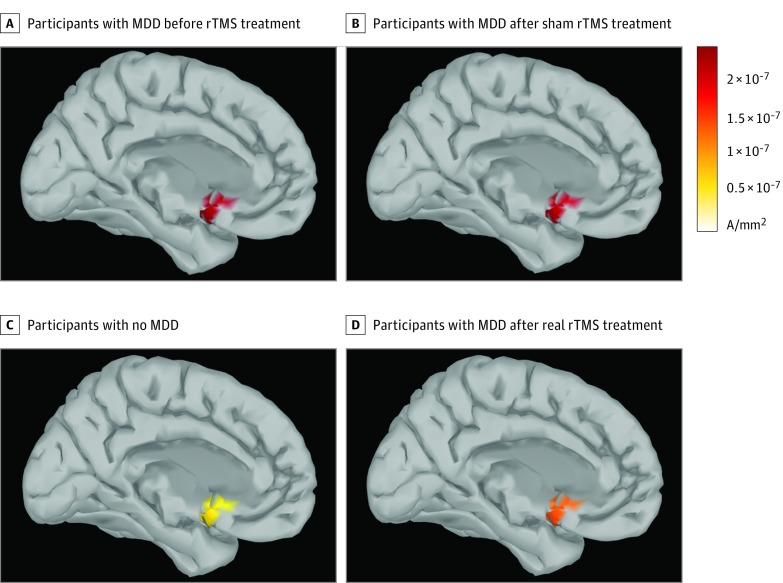
Current Density at the Subgenual Cingulate Cortex Region of Interest After Repetitive Transcranial Magnetic Stimulation (rTMS) Pulse MDD indicates major depressive disorder.

**Figure 5.**
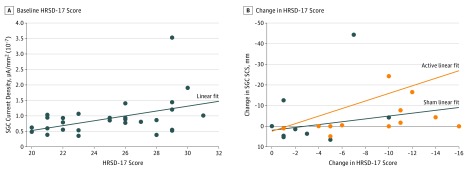
Association of Repetitive Transcranial Magnetic Stimulation Treatment With 17-Item Hamilton Rating Scale for Depression (HRSD-17) Scores A, Scores on HRSD-17 for participants with major depressive disorder before repetitive transcranial magnetic stimulation treatment were associated with current density at the subgenual cingulate cortex (SGC) (ρ = 0.41; *P* = .03). B, Changes on HRSD-17 scores for participants with major depressive disorder were associated with change in SGC significant current scattering (SCS) in participants with major depressive disorder receiving active (ρ = 0.58; *P* = .047) but not sham (ρ = 0.22; *P* = .54) repetitive transcranial magnetic stimulation treatment.

We then compared patients with MDD after active vs sham rTMS treatment (Figure 2B). Differences in mean (SD) current density between unilateral and bilateral rTMS treatment groups were not statistically significant (1.77 × 10^–7^ [4.63 × 10^–7^] μA/mm^2 ^vs 1.38 × 10^–7^ [2.43 × 10^–7^] μA/mm^2^; *z* = −1.00; *P* = .31). Therefore, we pooled these treatment groups to achieve better averaged neurophysiological signals and greater statistical power. Marked differences of current density, SCD, and SCS were found between the 2 experimental groups and were also localized in SGC-related voxels. These timing differences were also associated with the known TEP components (P60 milliseconds and P200 milliseconds). Mean (SD) current density in the SGC was higher for the sham group compared with the active rTMS group at 50 milliseconds (9.81 × 10^−8^ [4.26 × 10^−8^] μA/mm^2^ vs 1.87 × 10^−7^ [2.05 × 10^−7^] μA/mm^2^; *z* = 2.53; *P* = .01) and at 200 milliseconds (1.80 × 10^−7^ [1.10 × 10^−7^] μA/mm^2^ vs 1.10 × 10^−7^ [5.60 × 10^−8^] μA/mm^2^; *z* = −2.02; *P* = .04) after the TMS pulse. This comparison is also presented in Figure 4 as a region of interest activation around 200 milliseconds after stimulation. Mean (SD) SCD in the sham group was higher at 200 milliseconds compared with the active group (7.00 × 10^−7^ [7.51 × 10^−7^] μA/mm^2^ vs 1.57 × 10^−7^ [3.67^−7^ × 10^−7^] μA/mm^2^; *z* = −2.91; *P* = .004). Additionally, the SCS between the stimulation site (left DLPFC) and SGC was higher in the sham group than the active group at 200 milliseconds after the TMS pulse (0.74 [0.73] mm vs 0.20 [0.44] mm; *z* = −2.78; *P* = .006). Figure 5A shows the association of SGC source current density with HRSD-17 score in participants with MDD before rTMS treatment (ρ = 0.41; *P* = .03). The correlation shows that symptoms of depression were more pronounced when SGC current density was higher at 100 milliseconds after the TMS pulse. Figure 5B shows a correlation of the change in HRSD-17 score from baseline to post-rTMS treatment with change in SCS from baseline to post-rTMS treatment at approximately 100 milliseconds after the pulse. Patients with MDD showed a significant correlation between these measures after active treatment (ρ = 0.58; *P* = .047). The correlation between HRSD-17 change and SCS change in patients with MDD after sham treatment was weaker and not significant (ρ = 0.22; *P* = .54). The HRSD-17 score change demonstrated a stronger correlation with SCS change after active treatment compared with the correlation of SCS change after sham treatment. However, these 2 correlations were not significantly different (*z* = 0.877; *P* = .19).

## Discussion

This study’s results show that patients with MDD had higher-amplitude SGC-localized source measures and higher DLPFC-SGC effective connectivity compared with healthy controls. We found that SCD indexing had a high accuracy in discriminating patients with MDD from healthy controls and that the SGC-localized signal at approximately 100 milliseconds post-TMS pulse was correlated with depression severity before rTMS treatment. Moreover, the physiological signature of higher SGC-localized source measures in patients with MDD was attenuated after active rTMS treatment compared with patients with MDD who received sham rTMS. Finally, SGC-localized source measures (ie, SCS) were significantly correlated with improvement in depressive symptoms in the active treatment group but not in the sham group.

The higher SGC source activation of patients with MDD compared with healthy controls is in agreement with the limbic cortical dysregulation model,^37^ which suggests that the SGC is metabolically overactive in the depressed state^38^ and attenuated to lower levels with successful treatment.^6^ The attenuation of the SGC-localized SCD and SCS by rTMS treatment to values similar to those of untreated healthy controls, and the correlation of MDD symptom improvement with the change in left DLPFC-SGC SCS after treatment (Figure 5B) is supported by several converging lines of evidence that demonstrate reduction in SGC hyperactivity after responding to treatment, such as pharmacotherapy,^6,39^ deep brain stimulation,^4^ vagus nerve stimulation,^40^ and rTMS.^5,41,42,43,44^

The SGC-DLPFC interconnectivity may, in part, be governed by γ-aminobutyric acid (GABA)–ergic neurotransmission.^45^ Because rTMS is capable of modulating deep brain structure activity transsynaptically,^26^ the observed reduction of the effective connectivity signal (ie, SCS) between those 2 regions might be associated with the reconfiguration of these long-range connections. The SGC current density at approximately the 100 millisecond peak, its correlation with symptom severity at baseline (Figure 5A), and the correlation between symptom change and SCS signal change at approximately 100 milliseconds (Figure 5B) may also be associated with GABAergic neurotransmission because the evoked potential at approximately 100 milliseconds post-TMS pulse is associated with neuronal inhibitory processes.^46,47,48,49^ Using the EEG sensor distribution for localizing electrical generators in the cortex yields different possible spatial arrangements of source current generators. Incorporating some assumptions regarding brain anatomy and electrophysiological energetic constraints into the source localizing computation will restrict the multiplicity of possible solutions, but it will remain nonabsolute. Further, the immense spatial and temporal complexity of brain activity and the noisy nature of the EEG signal increase uncertainties when trying to solve this inverse problem.^50^ However, when TMS-EEG is applied, the timing and location of brain activation is constrained by the experimenter, reducing substantially the short latency inaccuracies of the source estimations. Additionally, as TMS was applied over the DLPFC, we can rely on prior knowledge regarding DLPFC connectivity and its association with our main region of interest, the SGC. This supports the potential of SGC activation after longer latencies following the TMS induction.

Apart from the advantages of using TMS-EEG to assess activation of localized generators in the brain mentioned earlier, we also implemented SCD and SCS statistical indexing over our data, which further mitigates erroneous source evaluations. On its own, simple signal averaging is a strong tool for reducing noise in our electrophysiological recordings. When applied over the averaged signal, SCD is far more statistically stringent by taking into account the whole distribution of the activation—making this indexing robust when dealing with signal outliers.^13^ The SCS takes the SCD approach a step further by accumulating the distances between significant sources in the SGC and the site of stimulation. Hence, the SCS computation estimates the effective connectivity between those 2 sites after stimulation. The methodological and computational steps we took to ensure our data quality and the fact that our a priori hypotheses were supported by our results were key in making our source estimations findings more deterministic and valid.

### Limitations

This study had some limitations. Our study design allowed only a between-participant rather than within-participant statistical inference, which is not ideal in cases that look at the potential effects of treatment. Despite this issue, each group in this study had an adequate number of participants. Moreover, the measured associations produced highly comparable values between the independent groups, enabling valid statistical inferences. It is important to note that to achieve adequate neurophysiological signals, the active treatment group was pooled from the bilateral rTMS treatment arm and the unilateral rTMS treatment arm (as depicted in Figure 1). However, the unilateral and bilateral treatment groups did not show statistical differences in EEG activations. As another limitation, patients with MDD in this study were receiving heterogeneous antidepressant pharmacotherapy. This fact may have confounded our clinical symptom assessments and EEG signal observations. However, the use of sham control treatment and the finding that only the active rTMS group demonstrated a correlation of the EEG signal with symptom improvement suggests that these changes may stem from the rTMS treatment itself rather than from concomitant pharmacotherapy. Moreover, the depression change score had a stronger correlation with SCS change after active treatment compared with the correlation after sham treatment. However, our sample size was likely not large enough to find a significant difference in these 2 correlations.

## Conclusions

In conclusion, this study demonstrated the usefulness of TMS-EEG and the SCD/SCS computation when investigating the association of SGC activation and DLPFC-SGC effective connectivity with MDD pathophysiology and clinical improvement. Left DLPFC rTMS may have improved MDD symptoms by altering the connectivity between the DLPFC and the SGC, likely via GABAergic neurotransmission. These findings support the hypothesis of SGC involvement in the pathophysiology and clinical improvement of MDD and also provide an objective biological target to differentiate mood states in MDD and to differentiate patients with MDD from healthy controls. Further research in larger patient populations and across different treatment modalities are warranted to further assess the diagnostic reliability and clinical usefulness of TMS-EEG in MDD.

## Notes

Supplement. **eTable. **Demographic Characteristics of Participants

## References

[1] Kessler RC, Berglund P, Demler O, ; National Comorbidity Survey Replication The epidemiology of major depressive disorder: results from the National Comorbidity Survey Replication (NCS-R). JAMA. 2003;289(23):-. doi:10.1001/jama.289.23.3095 12813115

[2] Rush AJ, Trivedi MH, Wisniewski SR, Acute and longer-term outcomes in depressed outpatients requiring one or several treatment steps: a STAR*D report. Am J Psychiatry. 2006;163(11):1905-1917. doi:10.1176/ajp.2006.163.11.1905 17074942

[3] Mayberg HS, Liotti M, Brannan SK, Reciprocal limbic-cortical function and negative mood: converging PET findings in depression and normal sadness. Am J Psychiatry. 1999;156(5):675-682. doi:10.1176/ajp.156.5.675 10327898

[4] Mayberg HS, Lozano AM, Voon V, Deep brain stimulation for treatment-resistant depression. Neuron. 2005;45(5):651-660. doi:10.1016/j.neuron.2005.02.014 15748841

[5] Fox MD, Buckner RL, White MP, Greicius MD, Pascual-Leone A Efficacy of transcranial magnetic stimulation targets for depression is related to intrinsic functional connectivity with the subgenual cingulate. Biol Psychiatry. 2012;72(7):595-603. doi:10.1016/j.biopsych.2012.04.028 22658708 PMC4120275

[6] Mayberg HS, Brannan SK, Tekell JL, Regional metabolic effects of fluoxetine in major depression: serial changes and relationship to clinical response. Biol Psychiatry. 2000;48(8):830-843. doi:10.1016/S0006-3223(00)01036-2 11063978

[7] Kennedy SH, Evans KR, Krüger S, Changes in regional brain glucose metabolism measured with positron emission tomography after paroxetine treatment of major depression. Am J Psychiatry. 2001;158(6):899-905. doi:10.1176/appi.ajp.158.6.899 11384897

[8] Kennedy SH, Konarski JZ, Segal ZV, Differences in brain glucose metabolism between responders to CBT and venlafaxine in a 16-week randomized controlled trial. Am J Psychiatry. 2007;164(5):778-788. doi:10.1176/ajp.2007.164.5.778 17475737

[9] Keedwell P, Drapier D, Surguladze S, Giampietro V, Brammer M, Phillips M Neural markers of symptomatic improvement during antidepressant therapy in severe depression: subgenual cingulate and visual cortical responses to sad, but not happy, facial stimuli are correlated with changes in symptom score. J Psychopharmacol. 2009;23(7):775-788. doi:10.1177/0269881108093589 18635699

[10] Mottaghy FM, Keller CE, Gangitano M, Correlation of cerebral blood flow and treatment effects of repetitive transcranial magnetic stimulation in depressed patients. Psychiatry Res. 2002;115(1-2):1-14. doi:10.1016/S0925-4927(02)00032-X 12165364

[11] Liston C, Chen AC, Zebley BD, Default mode network mechanisms of transcranial magnetic stimulation in depression. Biol Psychiatry. 2014;76(7):517-526. doi:10.1016/j.biopsych.2014.01.023 24629537 PMC4209727

[12] Nobler MS, Oquendo MA, Kegeles LS, Decreased regional brain metabolism after ECT. Am J Psychiatry. 2001;158(2):305-308. doi:10.1176/appi.ajp.158.2.305 11156816

[13] Casali AG, Casarotto S, Rosanova M, Mariotti M, Massimini M General indices to characterize the electrical response of the cerebral cortex to TMS. Neuroimage. 2010;49(2):1459-1468. doi:10.1016/j.neuroimage.2009.09.026 19770048

[14] Komssi S, Aronen HJ, Huttunen J, Ipsi- and contralateral EEG reactions to transcranial magnetic stimulation. Clin Neurophysiol. 2002;113(2):175-184. doi:10.1016/S1388-2457(01)00721-0 11856623

[15] Voineskos AN, Farzan F, Barr MS, The role of the corpus callosum in transcranial magnetic stimulation induced interhemispheric signal propagation. Biol Psychiatry. 2010;68(9):825-831. doi:10.1016/j.biopsych.2010.06.021 20708172

[16] Massimini M, Ferrarelli F, Huber R, Esser SK, Singh H, Tononi G Breakdown of cortical effective connectivity during sleep. Science. 2005;309(5744):2228-2232. doi:10.1126/science.1117256 16195466

[17] Rajji TK, Sun Y, Zomorrodi-Moghaddam R, PAS-induced potentiation of cortical-evoked activity in the dorsolateral prefrontal cortex. Neuropsychopharmacology. 2013;38(12):2545-2552. doi:10.1038/npp.2013.161 23820586 PMC3799076

[18] Ferrarelli F, Massimini M, Sarasso S, Breakdown in cortical effective connectivity during midazolam-induced loss of consciousness. Proc Natl Acad Sci U S A. 2010;107(6):2681-2686. doi:10.1073/pnas.0913008107 20133802 PMC2823915

[19] Casarotto S, Määttä S, Herukka S-K, Transcranial magnetic stimulation-evoked EEG/cortical potentials in physiological and pathological aging. Neuroreport. 2011;22(12):592-597. doi:10.1097/WNR.0b013e328349433a 21753711

[20] Railo H, Tuominen J, Kaasinen V, Pesonen H Dynamic changes in cortical effective connectivity underlie transsaccadic integration in humans. Cereb Cortex. 2017;27(7):3609-3617. doi:10.1093/cercor/bhw182 27365299

[21] Johnson JS, Kundu B, Casali AG, Postle BR Task-dependent changes in cortical excitability and effective connectivity: a combined TMS-EEG study. J Neurophysiol. 2012;107(9):2383-2392. doi:10.1152/jn.00707.2011 22323626 PMC3362246

[22] Ferrarelli F, Riedner BA, Peterson MJ, Tononi G Altered prefrontal activity and connectivity predict different cognitive deficits in schizophrenia. Hum Brain Mapp. 2015;36(11):4539-4552. doi:10.1002/hbm.22935 26288380 PMC6869713

[23] Blumberger DM, Maller JJ, Thomson L, Unilateral and bilateral MRI-targeted repetitive transcranial magnetic stimulation for treatment-resistant depression: a randomized controlled study. J Psychiatry Neurosci. 2016;41(4):E58-E66. doi:10.1503/jpn.150265 27269205 PMC4915938

[24] Hamilton M A rating scale for depression. J Neurol Neurosurg Psychiatry. 1960;23(1):56-62. doi:10.1136/jnnp.23.1.56 14399272 PMC495331

[25] World Medical Association World Medical Association Declaration of Helsinki: ethical principles for medical research involving human subjects. JAMA. 2013;310(20):2191-2194. doi:10.1001/jama.2013.281053. 24141714

[26] Rossini PM, Burke D, Chen R, Non-invasive electrical and magnetic stimulation of the brain, spinal cord, roots and peripheral nerves: basic principles and procedures for routine clinical and research application: an updated report from an IFCN Committee. Clin Neurophysiol. 2015;126(6):1071-1107. doi:10.1016/j.clinph.2015.02.001 25797650 PMC6350257

[27] Delorme A, Makeig S EEGLAB: an open source toolbox for analysis of single-trial EEG dynamics including independent component analysis. J Neurosci Methods. 2004;134(1):9-21. doi:10.1016/j.jneumeth.2003.10.009 15102499

[28] Oostenveld R, Fries P, Maris E, Schoffelen J-M FieldTrip: Open source software for advanced analysis of MEG, EEG, and invasive electrophysiological data. Comput Intell Neurosci. 2011;2011:156869. 21253357 10.1155/2011/156869 PMC3021840

[29] Rogasch NC, Sullivan C, Thomson RH, Analysing concurrent transcranial magnetic stimulation and electroencephalographic data: a review and introduction to the open-source TESA software. Neuroimage. 2017;147:934-951. doi:10.1016/j.neuroimage.2016.10.031 27771347

[30] Tadel F, Baillet S, Mosher JC, Pantazis D, Leahy RM Brainstorm: a user-friendly application for MEG/EEG analysis. Comput Intell Neurosci. 2011;2011:879716. doi:10.1155/2011/879716 21584256 PMC3090754

[31] Mazziotta J, Toga A, Evans A, A probabilistic atlas and reference system for the human brain: International Consortium for Brain Mapping (ICBM). Philos Trans R Soc Lond B Biol Sci. 2001;356(1412):1293-1322. doi:10.1098/rstb.2001.0915 11545704 PMC1088516

[32] Gramfort A, Papadopoulo T, Olivi E, Clerc M OpenMEEG: opensource software for quasistatic bioelectromagnetics. Biomed Eng Online. 2010;9:45. doi:10.1186/1475-925X-9-45 20819204 PMC2949879

[33] Pascual-Marqui RD Standardized low-resolution brain electromagnetic tomography (sLORETA): technical details. Methods Find Exp Clin Pharmacol. 2002;24(suppl D):5-12. 12575463

[34] Destrieux C, Fischl B, Dale A, Halgren E Automatic parcellation of human cortical gyri and sulci using standard anatomical nomenclature. Neuroimage. 2010;53(1):1-15. doi:10.1016/j.neuroimage.2010.06.010 20547229 PMC2937159

[35] Swets JA Measuring the accuracy of diagnostic systems. Science. 1988;240(4857):1285-1293. doi:10.1126/science.3287615 3287615

[36] Youden WJ Index for rating diagnostic tests. Cancer. 1950;3(1):32-35. doi:10.1002/1097-0142(1950)3:1<32::AID-CNCR2820030106>3.0.CO;2-3 15405679

[37] Mayberg HS Limbic-cortical dysregulation: a proposed model of depression. J Neuropsychiatry Clin Neurosci. 1997;9(3):471-481. doi:10.1176/jnp.9.3.471 9276848

[38] Drevets WC, Savitz J, Trimble M The subgenual anterior cingulate cortex in mood disorders. CNS Spectr. 2008;13(8):663-681. doi:10.1017/S1092852900013754 18704022 PMC2729429

[39] Drevets WC, Bogers W, Raichle ME Functional anatomical correlates of antidepressant drug treatment assessed using PET measures of regional glucose metabolism. Eur Neuropsychopharmacol. 2002;12(6):527-544. doi:10.1016/S0924-977X(02)00102-5 12468016

[40] Nahas Z, Teneback C, Chae J-H, Serial vagus nerve stimulation functional MRI in treatment-resistant depression. Neuropsychopharmacology. 2007;32(8):1649-1660. doi:10.1038/sj.npp.1301288 17203016

[41] Teneback CC, Nahas Z, Speer AM, Changes in prefrontal cortex and paralimbic activity in depression following two weeks of daily left prefrontal TMS. J Neuropsychiatry Clin Neurosci. 1999;11(4):426-435. doi:10.1176/jnp.11.4.426 10570754

[42] Nahas Z, Teneback CC, Kozel A, Brain effects of TMS delivered over prefrontal cortex in depressed adults: role of stimulation frequency and coil-cortex distance. J Neuropsychiatry Clin Neurosci. 2001;13(4):459-470. doi:10.1176/jnp.13.4.459 11748315

[43] Kito S, Fujita K, Koga Y Regional cerebral blood flow changes after low-frequency transcranial magnetic stimulation of the right dorsolateral prefrontal cortex in treatment-resistant depression. Neuropsychobiology. 2008;58(1):29-36. doi:10.1159/000154477 18781088

[44] Kito S, Hasegawa T, Koga Y Neuroanatomical correlates of therapeutic efficacy of low-frequency right prefrontal transcranial magnetic stimulation in treatment-resistant depression. Psychiatry Clin Neurosci. 2011;65(2):175-182. doi:10.1111/j.1440-1819.2010.02183.x 21414091

[45] Medalla M, Barbas H The anterior cingulate cortex may enhance inhibition of lateral prefrontal cortex via m2 cholinergic receptors at dual synaptic sites. J Neurosci. 2012;32(44):15611-15625. doi:10.1523/JNEUROSCI.2339-12.2012 23115196 PMC3523186

[46] Premoli I, Castellanos N, Rivolta D, TMS-EEG signatures of GABAergic neurotransmission in the human cortex. J Neurosci. 2014;34(16):5603-5612. doi:10.1523/JNEUROSCI.5089-13.2014 24741050 PMC6608220

[47] Rogasch NC, Daskalakis ZJ, Fitzgerald PB Cortical inhibition of distinct mechanisms in the dorsolateral prefrontal cortex is related to working memory performance: a TMS-EEG study. Cortex. 2015;64:68-77. doi:10.1016/j.cortex.2014.10.003 25461708

[48] Du X, Rowland LM, Summerfelt A, TMS evoked N100 reflects local GABA and glutamate balance. Brain Stimul. 2018;11(5):1071-1079. doi:10.1016/j.brs.2018.05.002 29759942 PMC6109427

[49] Loheswaran G, Barr MS, Zomorrodi R, Alcohol impairs N100 response to dorsolateral prefrontal cortex stimulation. Sci Rep. 2018;8(1):3428. doi:10.1038/s41598-018-21457-z 29467392 PMC5821878

[50] Pascual-Marqui RD, Esslen M, Kochi K, Lehmann D Functional imaging with low resolution brain electromagnetic tomography (LORETA): review, new comparisons, and new validation. https://www.uzh.ch/keyinst/NewLORETA/SomePapers/LorReview2.pdf. Accessed May 13, 2019. 12575492

