# Supplementary material for: Association of Repetitive Transcranial Magnetic Stimulation Treatment With Subgenual Cingulate Hyperactivity in Patients With Major Depressive Disorder: A Secondary Analysis of a Randomized Clinical Trial
Source: JAMA Netw Open. 2019 Jun 5;2(6):e195578. doi: 10.1001/jamanetworkopen.2019.5578 (PMC6551850; doi:10.1001/jamanetworkopen.2019.5578)
Supplement: Supplement. — eTable. Demographic Characteristics of Participants [file jamanetwopen-2-e195578-s001.pdf]

Supplementary Online Content

Hadas I, Sun Y, Lioumis P, et al. Association of repetitive transcranial magnetic stimulation treatment with subgenual cingulate hyperactivity in patients with major depressive disorder: a secondary analysis of a randomized clinical trial. *JAMA Netw Open*. 2019;2(6):e195578. doi:10.1001/jamanetworkopen.2019.5578

eTable. Demographic Characteristics of Participants

This supplementary material has been provided by the authors to give readers additional information about their work.

© 2019 Hadas I et al. *JAMA Network Open.*

eTable. Demographic Characteristics of Participants

	Healthy participants	MDD participants, before treatmenet	MDD participants after sham rTMS treatment	MDD participants after active rTMS treatment
**Age, Years mean (SD)**	37.03 (11.03)	39.13 (10.89)	45.66 (12.46)	47.32 (14.05)
**Sex, males**	15	15	9	9
**HRSD-17 score, mean (SD)**	-	24.80 (3.48)	19.82 (5.20)	17.37 (5.97)
**Medications**				
**SSRI**	-	14	11	12
**SNRI**	-	9	10	9
**Mirtazapine**	-	2	2	1
**Bupropion**	-	6	10	7
**TCA**	-	0	18	17
**MAOI**	-	1	2	3
**Benzodiazepine, n**	-	11	5	10
**Zopiclone, n**	-	4	5	1
**Antipsychotic, n**	-	7	9	8
**Mood stabilizer, n**	-	2	4	2

MDD, major depressive disorder; rTMS, repetitive transcranial magnetic stimulation; HRSD-17, 17-item Hamilton Rating Scale for Depression; MAOI, monoamine oxidase inhibitor; SNRI, serotonin and norepinephrine reuptake inhibitor; SSRI, selective serotonin reuptake inhibitor; TCA, tricyclic antidepressant.

© 2019 Hadas I et al. *JAMA Network Open.*
